# Out-of-Pocket Health Expenditure and Poverty: Evidence from a Dynamic Panel Threshold Analysis

**DOI:** 10.3390/healthcare9050536

**Published:** 2021-05-03

**Authors:** Abdalla Sirag, Norashidah Mohamed Nor

**Affiliations:** School of Business and Economics, Universiti Putra Malaysia, Serdang 43400 UPM, Malaysia; norashidah@upm.edu.my

**Keywords:** out-of-pocket health expenditure, poverty, poverty headcount, poverty gap, dynamic panel threshold

## Abstract

The current study investigated the association between out-of-pocket health expenditure and poverty using macroeconomic data from a sample of 145 countries from 2000 to 2017. In particular, it was examined whether the relationship between out-of-pocket health expenditure and poverty was contingent on a certain threshold level of out-of-pocket health spending. The dynamic panel threshold method, which allows for the endogeneity of the threshold regressor (out-of-pocket health expenditure), was used. Three indicators were adopted as poverty measures, namely the poverty headcount ratio, the poverty gap index, and the poverty gap squared index. At the same time, out-of-pocket health expenditure was measured as a percentage of total health expenditure. The results showed the validity of the estimated threshold models, indicating that only beyond the turning point, which was about 29 percent, that out-of-pocket health spending led to increased poverty. When heterogeneity was controlled for in the sample, using the World Bank income classification, the findings showed variations in the estimated threshold, with higher values for the low- and lower-middle-income groups, as compared to the high-income group. For the lower-income groups, below the threshold for out-of-pocket health expenditure, it had a positive or insignificant effect on poverty reduction, while it led to higher poverty above the threshold. Further, the sampled countries were divided into regions, according to the World Health Organization. Generally, improving health care systems through tolerable levels of out-of-pocket health expenditure is an inevitable step toward better health coverage and poverty reduction in many developing countries.

## 1. Introduction

As stated in the World Health Organization’s sustainable development goal 3.8, reaching universal health coverage and financial risk protection are important indicators to guarantee better healthy lives and higher well-being. In developing countries, health care resources are inadequate to guarantee that all individuals have equal access to necessary health needs. According to World Health Statistics [1], out-of-pocket health expenses can create financial hardship by forcing people to choose between health expenses and other necessities. Many studies have described out-of-pocket expenditure on health as catastrophic when it surpasses a certain threshold of a household’s consumption or income. Moreover, the proportion of the world’s population, which spent more than 10% of its household income on medical care, increased from 9.4% in 2000 to 12.7% in 2015, amounting to about US$927 million. The percentage of the population spending more than 25% of their family budget on health care also increased from 1.7% in 2000 to about 3% in 2015. The majority, or 87%, of the population that suffered huge out-of-pocket expenditures in 2015 were in middle-income countries. About 1 billion, or 12.9% of the population, were expected to spend at least 10% of their family budget on medical care by 2020 [1]. Financing health spending through out-of-pocket expenses has several severe consequences, such as pushing individuals and households into poverty. Most of the population who are pushed into extreme poverty, as a result of out-of-pocket healthcare expenditures, are located in less-developed countries. However, between 2000 and 2015, the number of people falling into poverty due to out-of-pocket healthcare spending (defined as US$1.90 per person per day) dropped from 123.9 million (2%) to 89.7 million (1.2%). This decline was in line with the decline in the total population living in extreme poverty. Additionally, out-of-pocket expenditure on health was the main source of economic disadvantages, such as low-income level. In particular, the increase in the world’s relative poverty, as a result of out-of-pocket health expenditure, was 110.9 million people in 2000 and 183.2 million people in 2015. Therefore, achieving universal health coverage remains an important challenge for many countries around the world. 

According to the World Health Organization and the World Bank [2], universal health coverage is defined as the extent to which public health services are designed to promote health, prevent disease, and provide high-quality treatment, rehabilitation, and palliative care sufficient to be effective while ensuring that the services that they provide will not expose the user to financial difficulties. The World Health Report [3] stated that to achieve universal health coverage, governments could take action in the following ways: allocate additional resources for health, lessen financial obstacles, and raise financial risk protection through pooling and prepayment; and ensure more equitable and efficient use of the available resources. Therefore, to have effective health systems, these systems must be efficiently financed by their host countries. In general, low-income countries have a higher share of private spending on health services than middle- and high-income countries, and most of the private health expenditures are out-of-pocket. 

Considerable numbers of studies have been carried out to investigate the impact on the impoverishment level of populations caused by out-of-pocket health spending [4,5,6,7,8]. It has been observed that poorer populations tend to incur higher catastrophic health spending and seek no, or lower quality of health care than less-poor populations [9,10]. One major issue in earlier out-of-pocket healthcare spending–poverty research concerned the reliance on the variation between specific poverty measures before and after out-of-pocket health expenses were included in total household consumption; as a primary empirical approach, see Wagstaff et al. [8]. Therefore, the previous literature on the impoverishing effects of out-of-pocket health spending has depended predominantly on survey-based or micro-level data of a specific country [11]. To date, however, few studies have investigated the association between out-of-pocket health expenditure and poverty using aggregated macro-level data [11], which is useful for providing cross-country analysis. It has also been argued that some households may spend up to a certain threshold of their budget (10% or 25%) on health care without affecting the resources left over to sustain their basic needs [12]. So far, very little attention has been paid to the idea that the relationship between out-of-pocket health spending and poverty is contingent on a certain threshold level.

There are several important areas where this study makes an original contribution to the out-of-pocket health spending literature. The main purpose of this study is to investigate the relationship between out-of-pocket health expenditure and poverty from a macroeconomic perspective. In particular, the hypothesized out-of-pocket health spending–poverty threshold nexus for 145 developed and developing countries over the period 2000 to 2017 is empirically examined. This study relies on Seo and Shin’s [13] endogenous panel threshold method, which has been recently introduced for its estimation procedure. Unlike most previous studies, poverty is measured by using three indicators, namely poverty headcount, poverty gap, and poverty gap squared. Finally, potential heterogeneity is controlled for in the sample.

The remainder of this paper proceeds as follows: Section 2 reviews the related empirical literature. Section 3 provides a brief overview of the methodology and data used in this study. Section 4 contains the results and discussion. Section 5 offers a conclusion to the paper and offers some thoughts regarding future studies.

## 2. Literature Review

The level of out-of-pocket health spending is mainly driven by variations in socioeconomic status, such as the income, age, and education level of households [14,15]. To provide policy responses, Xu et al. [4] investigated the determinants of catastrophic health spending. Their study defined out-of-pocket health payments as catastrophic if the spending on health exceeded 40% of household income. They argued that huge variations between countries regarding the percentage of households facing catastrophic out-of-pocket payments existed. Importantly, the coverage of health care services that required payment, the low ability to pay, and the nonexistence of health insurance were found to be among the essential factors that induced catastrophic health spending. Out-of-pocket payments were considered to be the major health care financing style in low-income countries and were mostly larger than government expenditure. The level of out-of-pocket expenditure tended to decline as income rose and when other forms of financing mechanisms increased [16]. Out-of-pocket healthcare payments are largely agreed to be a degrading, humble, and unsustainable manner to finance health care. The key determinants of catastrophic out-of-pocket health expenditure are poor economic conditions and low living standards [17,18]. Moreover, being part of a poor household can be one reason to pay for health care directly or out-of-pocket [7,8]. Excessive out-of-pocket payments may hinder access to health care, since they stop people from seeking necessary medical assistance and also deter them from receiving appropriate treatment [10,19].

Moreover, out-of-pocket expenditure on health care puts a huge burden on households and reduces their overall welfare [19], and Brown et al. [10] found that poorer households were less likely to experience catastrophic out-of-pocket payments since they avoided or delayed required health care. However, different evidence, as provided by Seeberg et al. [9], has suggested that low-income households sought medical care from less qualified providers, and, as a result, they faced catastrophic out-of-pocket spending. Another study by Ku et al. [20], conducted in Taiwan, argued that the implementation of National Health Insurance in the year 1995 contributed to reducing out-of-pocket health spending, especially for low-income households.

A large and growing body of literature has investigated the relationship between out-of-pocket health expenditure and poverty. Generally, out-of-pocket health spending was reported as a major reason for household impoverishment [5,6,21,22]. Poor health conditions were among the factors that contributed effectively to higher poverty rates, especially in developing countries such as Ghana [23]. Arsenijevic et al. [24] revealed that out-of-pocket health care payments were catastrophic for poor households and a leading cause of poverty in Serbia. Additionally, Koch et al. [22] showed that about 1% of households were pushed into poverty due to out-of-pocket health expenditure in Chile. Furthermore, Datta et al. [25] investigated the implications of out-of-pocket health expenditure, financial stress, and households’ impoverishment with non-communicable diseases. They found that non-communicable diseases induced higher medical expenditure, a greater probability of catastrophic out-of-pocket health spending, more financial stress, and a higher risk of impoverishment.

A study by Rashad and Sharaf [26] attempted to provide a new estimate for poverty that considered catastrophic out-of-pocket health expenditure in Egypt. Their approach relied on comparing the poverty gap and poverty line, both before and after the inclusion of catastrophic expenditures. They found that out-of-pocket payments led to 7.4% more households being pushed into poverty. Additionally, out-of-pocket health spending was strongly related to both the problems of inefficient and unequal access to health care; for example, low-income individuals who were not covered by social health insurance were more likely to have catastrophic out-of-pocket health spending than higher-income individuals covered by social health insurance [27]. Moreover, Van Minh et al. [7] noted that many poorer households faced catastrophic out-of-pocket health spending, and others were pushed into poverty. They found a modest impact from national health insurance on reducing impoverishment since it did not provide enough financial protection to households. Therefore, reducing the large share of out-of-pocket payments in health systems is a universal target for health system development. Eliminating out-of-pocket payments seems to be quite difficult, even within a health system offering free care. Kumara and Samaratunge [28] showed that free provision of health care did not stop the rising trend of out-of-pocket medical spending. However, as countries have accomplished high health coverage with well-organized health financing systems, they have found some significance in keeping certain out-of-pocket payments as incentives to have efficient health care performance. This may support the idea of the threshold effects of out-of-pocket health expenditure on poverty.

The existing literature on out-of-pocket health expenditure–poverty nexus has focused extensively on studying the issue in a single country setting, typically using survey data. Wagstaff et al. [8] and Wagstaff et al. [11] were among the few studies that used multi-country data and macroeconomic and health system indicators to investigate out-of-pocket health expenditure. Both of these studies indicated the existence of a large variation in out-of-pocket health spending across countries. Wagstaff et al. [11] revealed that out-of-pocket health expenditure at the USD 1.90 per person per day poverty line led to poverty, particularly in low-income countries. All of the studies reviewed so far have supported the hypothesis that the impoverishment impact of out-of-pocket health expenditure is asymmetric concerning certain factors such as the share of out-of-pocket health expenditure to the household’s budget or income. Yet, investigating the threshold effect of out-of-pocket health expenditure on poverty using cross-country and health system indicators remains a clear gap in the existing literature.

## 3. Methodology and Data

### 3.1. Model Specification and Estimation Technique

To examine the relationship between out-of-pocket health expenditure and poverty, the following model is specified:

(1) $$Pov_{it}=\beta OOP_{it}+\pi Z_{it}+\varepsilon_{it}$$

where $Pov_{it}$ refers to poverty, as measured by the poverty headcount, the poverty gap, and the poverty gap squared; $OOP_{it}$ is out-of-pocket health spending, as a percentage of total health expenditure; $Z_{it}$ is a vector of the explanatory variables; $GDP_{it}$ is the real income per capita; $GHE_{it}$ is government health expenditure, as a percentage of total health expenditure; $\varepsilon_{it}$ is an error term; i = 1, …, N denotes the country (N = 145 countries); and t = 1, …, T denotes the time, which is between 2000 and 2017.

As suggested in the existing literature, out-of-pocket health expenditure should be treated as an endogenous variable in its relationship with poverty. Out-of-pocket health payments can lead to higher population poverty, and at the same time, poverty may lead to increased financial risk and out-of-pocket health expenses. Therefore, it is critically important to control for the endogeneity of out-of-pocket health expenditure. Various threshold-estimating procedures can be used, such as Hansen’s [29] static panel method and Kremer’s et al. [30] dynamic panel technique. However, the methods mentioned above assume the exogeneity of the threshold regressor. Seo and Shin [13] suggested an alternative estimation procedure, which allows for the endogeneity of both regressors and the threshold variable. To estimate the non-linear relationship between out-of-pocket health expenditure and poverty in the sample of 145 countries (Table A3 in Appendix A shows the list of countries), the dynamic panel threshold technique was used, as suggested by Seo and Shin [13]. For that, let us consider the following threshold model:

(2) $$Pov_{it}()=u_{i}+\rho Pov_{it-1}+\beta_{1}OOP_{it}I\left(OOP_{it}\leq \gamma \right)+\beta_{2}OOP_{it}I\left(OOP_{it}>\gamma \right)+\pi Z_{it}+\varepsilon_{it}$$

where $u_{i}$ is the individual-specific effect, and $\varepsilon_{it}$ is the error term, which is assumed to be $\varepsilon_{it}$ ~ (0, $\sigma^{2}$). The indicator function I(⋅) indicates the regime or group, according to the threshold variable $OOP_{it}$, and γ denotes the impact of out-of-pocket health expenditure, depending on whether $OOP_{it}$ is below or above the threshold level. $Z_{it}$ contains a vector of the control variables, which are specified above. The impact of out-of-pocket health expenditure on poverty can be explained by ${\hat{\beta }}_{1}$(${\hat{\beta }}_{2}$) which denote the marginal effect of out-of-pocket health expenditure on poverty in the low (high) out-of-pocket health expenditure regime, i.e., when out-of-pocket health expenditure is below (above) the threshold. Normally, out-of-pocket health expenditure is relatively high in countries where health care coverage is low. Note that all the variables are transformed into natural logarithms as the coefficients are easier to interpret, and the data will most likely follow a normal distribution.

Equation (2) is estimated using the method of Seo and Shin [13], which allows for an endogenous threshold variable and regressors and uses the generalized method of moments (GMM) estimation technique as proposed by Arellano and Bond [31]. This technique comprises two steps: first, for a given threshold (γ), the coefficients (ρ, $\beta_{1}$, $\beta_{2}$, $\pi_{i}$) are estimated using the GMM estimator, as proposed by Arellano and Bond [31]. Second, the first step is repeated for the value of the threshold’s belonging in a strict subset of out-of-pocket health expenditure support, resulting in different estimates for each selected threshold. The threshold value (γ), which minimizes the objective function of the GMM estimator and its estimated parameters, is deemed to be the optimal threshold.

Parallel to other threshold estimating procedures, such as the Hansen [29] static method and the dynamic method of Kremer et al. [30], the Seo and Shin [13] technique has the advantage of allowing for the endogenous estimation of the threshold variable and any other regressors, such as the GDP per capita. This is applicable, in a practical sense, in this study because a higher poverty level may lead to greater out-of-pocket spending on health care due to the vulnerability and inadequacy of health coverage. To fit the properties of the GMM estimator, Equation (2) is estimated using three years of averaged data, namely from 2000 to 2017.

### 3.2. The Data

The World Bank frequently updates its definition of the poverty line as basic food, clothing, and housing costs change around the world. In 2015, the World Bank set the poverty line to be USD 1.90 per person per day, rather than USD 1.25 per person per day. The present study used three poverty measures based on the poverty line being set at USD 1.90 per person per day. First, the poverty headcount refers to the percentage of the population living below the national poverty line(s). Second, the poverty gap index of the World Bank is used. The poverty gap denotes the proportion by which the poor’s average income level dropped below the poverty line. Generally, a higher poverty gap indicates the increased severity of poverty in a given country. Finally, similarly to the second measure, the poverty gap squared index represents the extent of disparity among the poorer element of the population. The poverty headcount ratio, the poverty gap index, and the poverty gap squared index were retrieved from the World Bank PovcalNet [32].

Out-of-pocket health expenditure refers to individuals’ direct expenses to health care providers, excluding any prepayments for health services, such as taxes, insurance premiums, or contributions. Out-of-pocket health payments are part of health financing in all countries that rely on user fees and/or co-payments to rationalize the use of health services, advance health system efficiency, and improve the quality of their services [33]. Therefore, it is expected that a reasonable level of out-of-pocket health expenditure would be tolerable or insignificant to poverty reduction. Nonetheless, higher out-of-pocket spending, as a mean of health financing, is harmful to the eradication of poverty. Additionally, it can be argued that out-of-pocket health spending could lead to the impoverishment of households, while poverty of the population, in turn, could cause higher financial risk and out-of-pocket spending. The data for out-of-pocket health expenditure, as a percentage of total health expenditure, was obtained from the World Health Organization database [34]. Furthermore, the standard of living is an important factor that affects a nation’s poverty level; thereby, a higher quality of life is expected to reduce poverty. The growth of income per capita is one of the main sources for poverty reduction in many countries around the globe, since without an increase in economic growth, eradication of poverty remains a difficult task to be achieved [35,36,37,38,39]. Additionally, Škare and Družeta [40] argue that as economic growth takes place, poverty is reduced regardless of the level of income inequality. In this study, the real GDP per capita represented the statistical measure of the standard of living. The GDP per capita (constant USD 2010) was collected from the World Bank, World Development Indicators [41]. Lastly, the control variable of government health expenditure was measured as a share of general government expenditure, which may negatively or positively affect poverty. In general, the government is expected to affect poverty levels through its spending on important sectors such as education and health [42,43,44,45]. Thus, taking the impact of government expenditure on poverty reduction into consideration is important for modelling purposes. The data for government health expenditure were found in the World Health Organization database [34].

Table 1 presents the results of the descriptive statistics and correlation analysis. It is apparent from the table that the number of observations was around 870 for all the variables. What is interesting about the data in this table is that the poverty measures have large variations, as compared to the other variables, as they have the highest standard deviations. However, out-of-pocket health expenditure has a relatively low variation. These descriptive figures suggest that there are huge differences in poverty across nations, where variations concerning out-of-pocket health spending across countries exist; however, it remained comparatively lower. The results of the correlation matrix are summarized at the bottom of Table 1. Closer inspection of the correlation coefficients shows that there is high positive linear dependency among the three measures of poverty. Most importantly, the correlation among the explanatory variables remains low, which shows that the potential of collinearity is not of concern.

Normally, in using time series variables, testing the stationarity properties of the data is a common pretest. The results of the Im et al. [46] (IPS) unit root test, shown in Appendix A Table A1, revealed the variables to be stationary after taking the first-difference. Fortunately, the dynamic panel threshold used by our study relies on the Arellano and Bond [31] GMM estimator that runs the regression in first-difference.

## 4. Results and Discussion

Regression of the dynamic panel threshold analysis was used to predict the effect of out-of-pocket health expenditure on poverty. To assess the transition effect of out-of-pocket health spending on poverty, three aggregate indicators were used as poverty measures, namely poverty headcount, the poverty gap, and the poverty gap squared. Table 2 shows the results obtained from the analysis using the Seo and Shin [13] model.

When poverty headcount was used as the dependent variable, the results showed that the threshold of out-of-pocket health expenditure was about (ln3.378), or 29.3 percent, of total health expenditure, indicating that around 56 percent of the observations lay in the high out-of-pocket regime. Below the threshold, the coefficient ${\hat{\beta }}_{1}$ was negative and insignificant, indicating that out-of-pocket health expenditure did not influence poverty. However, above the threshold ${\hat{\beta }}_{2}$ was positive and statistically non-zero, which implied that any increase in out-of-pocket health spending would lead to more poverty. These findings were in line with the expectation that out-of-pocket health spending beyond a certain threshold would contribute to greater poverty. The lagged-dependent variable of poverty headcount was positive and statistically different from zero, signifying some persistence in poverty across countries. The results revealed a negative and significant relationship between the GDP per capita and the poverty headcount. This indicated that with continuous increases in the level of income, poverty was further reduced. There was no evidence that government health expenditure influenced poverty headcount.

When the poverty gap was used as the dependent variable, the estimated out-of-pocket threshold parameter was about (ln3.390) or 29.7 percent of total health expenditure, with approximately 55 percent of the observations in the high-out-of-pocket regime. The findings revealed a negative and statistically insignificant relationship between out-of-pocket health spending and the poverty gap below the threshold. In contrast, a positive and significant relationship was found above the threshold. These outcomes suggested that with higher out-of-pocket health expenditure that, beyond a certain level, poverty would increase. Moreover, the coefficient of the lagged-dependent variable of the poverty gap was shown to be positive and significant. There was a significant negative and significant association between the income per capita and the poverty gap, which suggested the importance of improving income in eliminating poverty. However, it was found that government health expenditure had no evident effect on the poverty gap.

Turning to the results of the poverty gap squared, when used as a dependent variable, the findings showed that the threshold of out-of-pocket health expenditure was about (ln3.390), or 29.7 percent, which indicated that about 55 percent of the observations were above the threshold of out-of-pocket health spending. It was found that ${\hat{\beta }}_{1}$ had no significant impact on the poverty gap squared, while ${\hat{\beta }}_{2}$ appeared to be positive and statistically significant. These findings implied that any additional out-of-pocket health spending beyond the threshold point led to an increased poverty gap squared. The lagged-dependent variable’s dynamic term was shown to be positively related to its current poverty gap squared. Moreover, it was found that the GDP per capita coefficient was negative and significantly associated with the poverty gap squared. A possible implication was that countries with a higher level of income were more likely to reduce poverty. Nevertheless, it was found that there was no change in the poverty gap squared associated with changes in government expenditure on health.

To summarize, above the threshold point, higher out-of-pocket health expenditure led to increased poverty. In addition, GDP per capita was an important factor that resulted in higher poverty reduction. At the same time, government health expenditure had no significant impact on poverty. To check the soundness of the estimated poverty threshold models, the results of the linearity test that had the null of no threshold effects were conducted. For the poverty headcount and poverty gap, it was found that the bootstrap *p*-values were 0.06 and 0.04, respectively, suggesting the existence of threshold effects. However, the bootstrap *p*-value for the poverty gap squared model was 0.2, indicating a non-threshold effect.

To control for variation across countries, the World Bank’s income classifications were relied upon, as well as the use of a group dummy. Table 3 shows the dynamic panel results with the endogenous threshold regressor controlling for low-income and lower-middle-income countries. When poverty headcount was the dependent variable, the estimated out-of-pocket health spending thresholds for low- and lower-middle-income countries were (ln3.292), or 26.9 percent, and (ln3.877), or 48.3 percent, of total health expenditure, respectively. These thresholds indicated that approximately 83 percent of the observations were in the high-out-of-pocket regime, in the case of low-income countries, whereas about 41.5 percent of the observations were in the upper regime of out-of-pocket health expenditure in lower-middle-income countries. Below the thresholds, it was found that out-of-pocket health expenditure had negative and significant effects on poverty headcount. However, beyond the thresholds, out-of-pocket health expenditure had positive and statistically meaningful effects on poverty. Earlier findings indicated that financing health expenditure through the out-of-pocket mode helped to reduce poverty initially. However, with further spending, the effect became a poverty-increasing factor. The results revealed that the dynamic term of poverty headcount was positive and statistically significant in both the sampled low and lower-middle-income countries. Importantly, negative and significant income per capita effects on poverty headcount were found in both income groups. This particular result suggested that improvement in the standard of living was an influential factor contributing to poverty reduction in the sampled less-developed countries, while there was evidence of a negative and positive relationship between government expenditure on health and poverty headcount in the sampled low- and lower-middle-income countries, respectively, suggesting that it led to reduced poverty in low-income and higher poverty as observed in the lower-middle-income group, as a result of government health spending.

When poverty was measured using the poverty gap, it was found that the out-of-pocket thresholds were (ln2.282), or 9.8 percent, and (ln3.679), or 39.6 percent, of total health expenditure, for the sampled low- and lower-middle-income countries, respectively. These turning points showed that nearly 96 percent of the observations were in the upper regime of out-of-pocket health spending for the sampled low-income countries. For the sampled lower-middle-income countries, around 59 percent of the observations were in the upper regime of out-of-pocket health spending. The results showed that the effect of out-of-pocket health spending on the poverty gap, below the threshold, was negative and significant for both low- and lower-middle-income countries. However, above the threshold, out-of-pocket health spending had positive and statistically meaningful effects on poverty, with a higher magnitude in the lower-middle-income group. The previous results suggested that out-of-pocket health expenditure lessened poverty initially but increased poverty eventually. Moreover, the results revealed that the lagged-dependent variable of the poverty gap was positive and significant in both the sampled low- and lower-middle-income countries. Notably, a negative and significant relationship was found between per capita income and the poverty gap in both income groups, suggesting that a higher standard of living contributed effectively to poverty reduction in the sampled less-developed countries. Lastly, the relationship between government health expenditure and the poverty gap was negative in the sample low-income and positive in the sampled lower-middle-income countries, but it was insignificant in the sampled lower-middle-income countries.

When the poverty gap squared was the dependent variable, the results showed that the thresholds of out-of-pocket health expenditure for both groups were (ln3.218), or 24.9 percent, and (ln3.877), or 48.3 percent, which indicated that about 83 percent and 41.5 percent of the observations were above the threshold of out-of-pocket health spending for both the sampled low- and lower-middle-income countries, respectively. Specifically, it was found that ${\hat{\beta }}_{1}$ had a negative and insignificant effect on the poverty gap squared in the low-income group, but it had a significant negative effect on poverty in the sampled lower-middle-income countries. Nevertheless, it was found that ${\hat{\beta }}_{2}$ had a positive and statistically significant effect on the poverty gap squared in both of the sampled groups of countries. These findings denoted that further out-of-pocket health expenditure, beyond the threshold, led to a higher poverty gap squared in both groups. The dynamic term of the poverty gap squared was positive and significantly related to its current level in both samples. Additionally, it was found that the coefficient of per capita income was negative and significantly connected to the poverty gap squared in both the sampled low- and lower-middle-income countries, implying that countries with a higher level of income were more likely to have lower poverty rates. However, it was found that government health expenditure was negatively related to the poverty gap squared in the sampled low-income countries. In contrast, it had no significant effect on poverty in the lower-middle-income group.

Beyond the threshold point, a greater level of out-of-pocket health expenditure led to a higher poverty rate, while the effect of income per capita was negatively related to different poverty measures. However, the effect of government health expenditure on poverty was ambiguous. The bottom of Table 3 shows the linearity test outcomes that had the null hypothesis of no threshold. For the sampled low- and lower-middle-income countries, it was found that all of the bootstrap *p*-values for the three models of poverty were less than 0.05, which suggested the validity of the threshold effects of out-of-pocket health spending on poverty.

Table 4 demonstrates the results obtained from the use of Seo and Shin’s [13] dynamic panel threshold analysis, controlling for both upper-middle-income and high-income countries. The results revealed that the estimated out-of-pocket health spending threshold was (ln3.750), or 42.5 percent, of total health expenditure for the three poverty models in the sampled upper-middle-income countries. The threshold indicated that nearly 27 percent of the observations were in the upper out-of-pocket regime in the sampled upper-middle-income countries. As shown in Table 4, the outcomes indicated that out-of-pocket health spending had positive and significant effects on poverty below the threshold. However, above the threshold, out-of-pocket health spending had no significant effects on poverty in the sampled upper-middle-income countries. The former results showed that out-of-pocket health expenditure led to increased poverty initially. However, beyond a certain level, poverty became irresponsive to changes in out-of-pocket health spending in the sampled upper-middle-income countries. The reported results show that the lagged-dependent variable of poverty was positive and statistically significant in two out of the three models in the upper-middle-income sample. Notably, the relationship between the income per capita and poverty was negative and significant, indicating that a higher standard of living was a prominent element that lessened poverty in the sampled upper-middle-income nations. However, there was a positive relationship between government spending on health and poverty in the two models, suggesting that a higher level of poverty might result from higher government spending on health.

Table 4 shows the results of the estimated poverty models for the sampled high-income countries. The out-of-pocket threshold values were (ln2.218), or 9.2 percent, (ln3.106), or 22.3 percent, and (ln3.074), or 21.6 percent, of total health expenditure, for the three models, respectively. The threshold points illustrated that about 98.7 percent, 41.2 percent, and 43 percent of the observations were in the upper regime of out-of-pocket health spending for the three measures of poverty, respectively. (It is worth mentioning that the thresholds appeared to be invalid due to the failure of rejecting the null hypothesis of no threshold in the estimated models for the sampled high-income countries.) The findings indicated a positive relationship between out-of-pocket health expenditure and the three measures of poverty below the thresholds. The relationship between the two variables was inconsistent since it was mostly insignificant from a statistical point of view. The preceding effects proposed that greater out-of-pocket expenditure on health increased poverty in relatively developed nations. A positive and significant lagged-dependent variable was found for the poverty models in high-income countries. Moreover, there was a negative but mostly insignificant relationship between per capita income and poverty in two out of the three estimated models, which suggested that the higher standard of living in developed nations contributed less to poverty reduction. Nonetheless, a positive relationship between government health expenditure and the poverty headcount and poverty gap models was found, whereas the effect was insignificant in the poverty gap squared model.

To ensure the accuracy of the estimated threshold models for the upper-middle-income and high-income groups, the results of the linearity test are shown in Table 4. For the sampled upper-middle-income countries, the bootstrap *p*-values were 0.04, 0.0, and 0.08, for the poverty headcount, poverty gap, and poverty gap squared models, respectively, suggesting the validity of the threshold effects between out-of-pocket health spending and poverty. However, for the sampled high-income countries, the bootstrap *p*-values for all of the models were greater than 0.1, indicating the nonexistence of a threshold effect between out-of-pocket health spending and poverty in the high-income group.

An additional analysis was conducted to control for heterogeneity across the sampled countries, using the World Health Organization’s regional classifications as the criteria. A dummy variable for each of the WHO’s six regions was used, namely Africa, the Americas, Eastern Mediterranean, Europe, South-East Asia, and Western Pacific. Table 5 and Table 6 present the threshold regression analysis results after controlling for the six regions. Starting with the African region, it was found that the estimated thresholds were between (ln3.694), or 40.2 percent, and (ln3.851), or 48.04 percent. From the results in Table 5, it was apparent that out-of-pocket health expenditure had a positive and statistically significant effect on poverty, both below and above the turning points, which suggested that out-of-pocket health expenditure led to increased poverty in many African countries. Regarding the results for the control variables, it was found that the lagged-dependent variable and government expenditure were positively related to poverty. However, income per capita was negative and significantly affected poverty in the African states, pointing toward the importance of economic growth and improving the population’s welfare in reducing poverty.

Turning to the empirical evidence for the Americas region, the results indicated that the threshold’s values were (ln2.487), or 12 percent, and (ln2.662), or 14.3 percent. From Table 5, it can be observed that there was evidence of non-linear associations between out-of-pocket health expenditure and poverty. Below the threshold, it was found that ${\hat{\beta }}_{1}$ was negative and significantly associated with the poverty gap and poverty gap squared, whereas ${\hat{\beta }}_{2}$ appeared to be positive and statistically different from zero. Looking at the results for the other independent variables, a similar conclusion was found to the findings of earlier models. When the same poverty models were estimated for the Eastern Mediterranean region, it was observed that the effect of out-of-pocket health spending on poverty was statistically insignificant for both ${\hat{\beta }}_{1}$ and ${\hat{\beta }}_{2}$, although the three models rejected the null hypothesis of no threshold effect. Moreover, most of the explanatory variables were not different from zero.

Regarding the results for the European region, as shown in Table 6, the different values of the threshold ranged from (ln2.331), or 10.3 percent, to (ln3.444), or 31.3 percent, of total health expenditure. However, the null hypothesis of no threshold effect could not be rejected for the three models. The outcomes revealed that, below the threshold, there was a positive relationship between out-of-pocket health expenditure and poverty. However, the coefficients above the threshold levels were inconsistent since they appeared to be negatively related to poverty headcount and poverty gap squared but positively correlated to the poverty gap. In contrast, the findings of the lagged-dependent variable and government expenditure were found to be positive and statistically significant, whereas the GDP per capita had a negative and significant effect on poverty, which supported the essential role played by higher income levels on poverty reduction in the European region.

The results for the South-East Asian region are presented in Table 6. The estimated threshold values were (ln2.661), or 14.3 percent, and (ln3.028), or 20.7 percent, of total health expenditure. The results showed an insignificant relationship between out-of-pocket health expenditure and poverty, both below and above the thresholds. However, the upper regime coefficients were positive, showing potential inverse effects of out-of-pocket health expenditure on poverty. The poverty level in the region showed some persistence due to the significance of lagged-poverty. However, the other explanatory variables were found to be insignificantly associated with poverty in the South-East Asian region.

In the final part of the analysis, the threshold model results for the Western Pacific region are shown in Table 6. It is interesting to note that the findings for this region revealed the lowest threshold levels, as compared to the models for the other regions. These thresholds ranged from (ln1.024), or 2.8 percent, to (ln2.121), or 8.3 percent. Unsurprisingly, therefore, the coefficients in the lower regime of out-of-pocket spending were positively related to various poverty measures, while the coefficients in the upper regime had a negative sign. This result indicated that higher out-of-pocket health expenditure both increased poverty below the threshold and reduced poverty above the threshold. The previous result may have been due to the low level of out-of-pocket health expenditure in the Western Pacific region. The dynamic terms of poverty appeared to be significant in the two models. Moreover, the impact of income per capita on poverty was negative, which suggested a positive association between the income level and poverty reduction. Regarding government expenditure on health, there was slight evidence that it harmed the poverty headcount.

Table A2 in Appendix A shows the results of the robustness analysis after we included additional variables such as corruption index and income inequality. The main findings regarding the threshold effect of out-of-pocket health spending on poverty remained similar to the earlier results.

A strong relationship between out-of-pocket health payments and poverty has been reported in the previous literature. Similarly, this study found a significant association between these variables. However, the results provided more evidence of a threshold impact of out-of-pocket health spending on poverty. A moderate level of out-of-pocket health expenditure can be beneficial to poverty reduction, whereas beyond a threshold, out-of-pocket health expenditure fuels poverty. Previous studies have highlighted this issue, mainly in the context of a single country, except for Wagstaff et al. [8] and Wagstaff et al. [11]. Nevertheless, the present study has contributed to the existing literature in that regard by using macroeconomic data from multiple countries. 

## 5. Conclusions

Reducing out-of-pocket spending on health is not only essential to protect individuals and households against any financial risk, but it is an inevitable element to eradicate poverty. The present study was designed to determine the effects of out-of-pocket health expenditure on poverty, using cross-country macroeconomic data. The use of various poverty measures in this study was also an additional distinguishing feature, as compared to the reviewed previous studies. Specifically, the threshold relationship between out-of-pocket health spending and poverty was tested on data from 145 countries, using an appropriate econometric technique. In addition, any potential heterogeneity regarding out-of-pocket health expenditure and poverty was controlled for by running different regressions, according to the World Bank’s income groups and the World Health Organization’s regions.

The findings obtained by the dynamic panel threshold regression revealed the existence of a threshold effect of out-of-pocket health spending on poverty. Below the threshold of approximately 29%, out-of-pocket health expenditure made no significant difference to the level of poverty. However, further increases in out-of-pocket health expenditure, as a percentage of total health expenditure, led to greater poverty above the threshold. Beyond the threshold mentioned above, a 1% increase in out-of-pocket health expenditure, as a percentage of total health expenditure, led to around 1.8%, 2%, and 2% increases in the poverty headcount, the poverty gap index, and the poverty gap squared index, respectively. These results supported the idea that in countries where out-of-pocket health expenditure is higher than the threshold, which is mainly in the less-developed world, individuals are pushed into poverty due to higher health financial risk.

When income differences across countries were taken into consideration, this study’s results showed relatively higher turning points in the sampled low- and middle-income countries rather than in the sampled high-income nations. In particular, in low- and lower-middle-income countries, the effects of out-of-pocket health spending helped to reduce various poverty measures. Above the thresholds, however, out-of-pocket health expenditure increased poverty. In the case of the sampled upper-middle- and high-income states, out-of-pocket health expenditure appeared to be harmful to poverty below the thresholds, while it was insignificant beyond the thresholds. Note that the results of the upper regime in the upper-middle-income groups showed relatively fewer observations (countries) in the higher out-of-pocket-regime, whereas the threshold effects were invalid in the high-income sample. The principal empirical implication of these outcomes is that in countries where the share of out-of-pocket health expenditure to total health expenditure is comparatively high, poorer household health most likely would lead to higher poverty.

To investigate the relationship between out-of-pocket health expenditure and poverty, the sampled countries were divided according to the World Health Organization’s regions. The results revealed that in four out of the six regions, the effects of out-of-pocket health spending on poverty were contingent on certain threshold levels. This showed that below the threshold, out-of-pocket health spending was negatively related to poverty, while it was positively correlated to poverty above the threshold, although the coefficients in two of the regions were not significant. The findings showed relatively low turning points regarding the European and Western Pacific regions, and out-of-pocket health expenditure was positive and negative, both below and above the threshold, respectively. In the European region, there were no threshold effects detected. However, out-of-pocket health expenditure seemed to positively impact poverty reduction, especially above the threshold in the Western Pacific region.

One of the most significant findings to emerge from this study was that the lagged-dependent variables positively influenced various poverty measures, indicating that poverty was affected by its previous level. Overall, this finding strengthened the idea that poorer households are most likely to remain in poverty compared to non-poor households. The current findings support the relevance of the living standard to poverty reduction. These findings have significant implications for understanding how the improvement of income per capita is translated to poverty reduction, while low income causes more poverty. Taken together, these findings provide support for the concept of the poverty trap. In particular, it is most likely that poorer nations with fairly low standards of living will remain in extreme poverty, on the one hand. On the other hand, improved living standards for poor countries may help them to escape the poverty trap [47]. Regarding the effect of health expenditure by governments on poverty, the findings of this study were uncertain.

The findings of this study have some important implications for future practice. The huge variations in poverty levels across the globe necessitate the significance of reprioritizing economic policies to make poverty and income inequality reduction the top item on each nation’s economic agenda. Moreover, there is an urgent need for fast structural reforms for health care systems, especially in the post-COVID-19 era. In particular, governments are responsible for eliminating any financial risk associated with health uncertainty, thus reducing excessive out-of-pocket health expenditure. Greater investment in health-related infrastructure will increase access to health care and reduce the burden of catastrophic out-of-pocket expenditure for populations, which may help to eliminate any poverty associated with financial hardship and out-of-pocket health payments. For future studies, alternative poverty lines, other than the level of USD 1.90 per person per day, might be used for comparison, since this level only accounts for extreme poverty, which suits developing nations. Lastly, future studies that aim to estimate country-specific out-of-pocket payment thresholds are recommended to examine national poverty reduction policies.

## Figures and Tables

**Table 1:** Descriptive statistics and correlation.

Variable	Obs	Mean	Std. Dev.	Min	Max	
$PHC_{it}$	870	−3.366	2.363	−9.924	−0.050	
$PG_{it}$	870	−4.483	2.420	−11.774	−0.415	
$PG_{it}^{2}$	870	−5.243	2.509	−13.149	−0.701	
$OOP_{it}$	870	3.315	0.848	−2.355	4.438	
$GDP_{it}$	870	8.314	1.461	5.315	11.5999	
$GHE_{it}$	870	2.186	0.514	0.318	3.518	
Correlation Matrix						
	$PHC_{it}$	$PG_{it}$	$PG_{it}^{2}$	$OOP_{it}$	$GDP_{it}$	$GHE_{it}$
$PHC_{it}$	1					
$PG_{it}$	0.9796	1				
$PG_{it}^{2}$	0.9371	0.9870	1			
$OOP_{it}$	0.1618	0.1403	0.1239	1		
$GDP_{it}$	−0.8070	−0.7287	−0.6489	−0.2744	1	
$GHE_{it}$	−0.4598	−0.4124	−0.3624	−0.3754	0.5696	1

Note: obs, St. Dev., Min, and Max, denote observation, standard deviation, minimum, and maximum, respectively. PHC, PG, and PG^2^ refer to poverty headcount, poverty gap, and poverty gap squared, respectively. All the variables are expressed in logarithmic form.

**Table 2:** Dynamic panel threshold results—all countries.

Variable	Head-Count	P-Gap	P-Gap^2^
Threshold (γ)	3.378 ***	3.390 ***	3.390 ***
	−0.169	−0.183	−0.183
Out-of-pocket			
${\hat{\beta }}_{1}$	−0.675	−0.703	−0.392
	−0.425	−0.479	−0.577
${\hat{\beta }}_{2}$	1.796 **	1.988 **	2.001 **
	−0.724	−0.831	−0.98
$Pov_{it-1}$	0.277 ***	0.267 ***	0.240 ***
	−0.073	−0.078	−0.075
$GDP_{it}$	−2.009 ***	−2.214 ***	−2.282 ***
	−0.211	−0.266	(0.306)
$GHE_{it}$	−0.019	−0.005	0.037
	−0.068	−0.079	−0.099
Upper regime (%)	55.86	55.17	55.17
Bootstrap (*p*-value)	0.06	0.04	0.2
N	145	145	145
No. moment conditions	32	32	32

Note: ***, ** denote 1%, 5%, respectively. Between ( ) are robust standard errors. N refers to the number of countries.

**Table 3:** Dynamic panel threshold results—controlling for income differences.

Variable	Low-Income Countries			Lower-Middle-Income Countries		
	Head-Count	P-Gap	P-Gap^2^	Head-Count	P-Gap	P-Gap^2^
Threshold (γ)	3.292 ***	2.282 ***	3.218 ***	3.877 ***	3.679 ***	3.877 ***
	−0.133	−0.051	−0.107	−0.008	−0.023	−0.016
Out-of-pocket						
${\hat{\beta }}_{1}$	−0.255 ***	−0.701 ***	−0.203	−0.541 ***	−0.875 ***	−0.915 ***
	−0.05	−0.187	−0.155	−0.132	−0.161	−0.138
${\hat{\beta }}_{2}$	0.358 ***	0.905 ***	0.821 ***	5.171 ***	2.856 ***	3.745 ***
	−0.055	−0.163	−0.116	−0.674	−0.195	−0.733
$Pov_{it-1}$	0.0416 ***	0.368 ***	0.250 ***	0.381 ***	0.424 ***	0.304 ***
	−0.029	−0.03	−0.05	−0.025	−0.023	−0.019
$GDP_{it}$	−0.759 ***	−1.084 ***	−1.492 ***	−1.710 ***	−1.918 ***	−2.485 ***
	−0.109	−0.12	−0.309	−0.114	−0.112	−0.122
$GHE_{it}$	−0.107 ***	−0.168 ***	−0.184 ***	0.099 **	0.045	0.052
	−0.015	−0.046	−0.04	−0.049	−0.047	(0.106)
Upper regime (%)	83.3	96.2	83.3	41.5	58.9	41.5
Bootstrap (*p*-value)	0	0	0	0.04	0	0.02
N	145	145	145	145	145	145
No. moment conditions	32	32	32	32	32	32

Note: ***, ** denote 1%, 5%, respectively. Between ( ) are robust standard errors. N refers to the number of countries.

**Table 4:** Dynamic panel threshold results—controlling for income differences (continue).

Variable	Upper-Middle-Income Countries			High-Income Countries		
	Head-Count	P-Gap	P-Gap^2^	Head-Count	P-Gap	P-Gap^2^
Threshold (γ)	3.750 ***	3.750 ***	3.750 ***	2.218 ***	3.106 ***	3.074 ***
	−0.056	−0.111	−0.152	−0.276	−0.06	−0.038
Out-of-pocket						
${\hat{\beta }}_{1}$	1.754 ***	2.032 ***	2.434 ***	6.256	2.988 ***	3.452 ***
	−0.169	−0.181	−0.214	−8.941	−0.942	−0.832
${\hat{\beta }}_{2}$	−2.243	−1.824	−2.354	−5.029	−2.211	−3.511 **
	−1.931	−1.492	−1.6	−8.969	−1.396	−1.457
$Pov_{it-1}$	0.215 ***	0.076 ***	−0.031	0.365 ***	0.476 ***	0.413 ***
	−0.018	−0.028	−0.035	−0.028	−0.044	−0.031
$GDP_{it}$	−2.762 ***	−2.924 ***	−2.953 ***	−0.558 ***	−0.394	−0.064
	−0.17	−0.315	−0.337	−0.138	−0.25	−0.243
$GHE_{it}$	0.198 ***	0.167 **	0.032	0.886 ***	0.821 **	0.245
	−0.048	−0.08	−0.076	−0.218	−0.337	(0.321)
Upper regime (%)	27.1	27.1	27.1	98.7	41.2	43.4
Bootstrap (*p*-value)	0.04	0	0.08	0.86	0.18	0.62
N	145	145	145	145	145	145
No. moment conditions	32	32	32	32	32	32

Note: ***, ** denote 1%, 5%, respectively. Between ( ) are robust standard errors. N refers to the number of countries.

**Table 5:** Dynamic panel threshold results—controlling for WHO regions.

Variable	African Region			Region of the Americas			Eastern Mediterranean Region		
	Head-Count	P-Gap	P-Gap^2^	Head-Count	P-Gap	P-Gap^2^	Head-Count	P-Gap	P-Gap^2^
Threshold (γ)	3.694 ***	3.851 ***	3.733 ***	2.487 ***	2.662 ***	2.662 ***	3.911 ***	3.911 ***	3.096 ***
	−0.044	−0.036	−0.099	−0.019	−0.026	−0.02	−0.419	−0.533	−0.046
Out-of-pocket									
${\hat{\beta }}_{1}$	0.091	0.292 ***	0.370 ***	−8.875	−8.543 ***	−10.718 ***	1.089	−0.022	76.289
	−0.061	−0.082	−0.102	−5.602	−1.349	−3.287	−2.625	−1.819	−498.56
${\hat{\beta }}_{2}$	1.822 ***	1.124 ***	0.474 *	9.818 *	9.858 ***	12.284 ***	6.945	6.179	−79.308
	−0.11	−0.278	−0.244	−5.549	−0.27	−3.387	−15.701	−12.368	−534.72
$Pov_{it-1}$	0.193 ***	0.256 ***	0.341 ***	0.087 ***	0.169 ***	0.259 ***	0.724	0.996	1.522
	−0.022	−0.027	−0.028	−0.024	−0.038	−0.039	−0.546	−0.606	−1.437
$GDP_{it}$	−0.443 ***	−0.691 ***	−0.678 ***	−2.550 ***	−2.671 ***	−2.219 ***	−3.627	−3.255	−1.691
	−0.064	−0.078	−0.096	−0.094	−0.251	−0.23	−3.173	−3.394	−4.908
$GHE_{it}$	0.158 ***	0.112 ***	0.069 *	0.063	0.184 ***	0.319 ***	3.193 *	2.827 *	3.498
	−0.029	−0.036	−0.036	−0.092	−0.067	−0.075	−1.788	−1.484	(20.649)
Upper regime (%)	48.02	36.5	42.9	98.7	93.6	93.6	66.7	66.7	95.2
Bootstrap (*p*-value)	0.04	0.1	0.8	0	0	0	0	0	0
N	145	145	145	145	145	145	145	145	145
No. moment conditions	32	32	32	32	32	32	32	32	32

Note: ***, **, * denote 1%, 5%, 10%, respectively. Between ( ) are robust standard errors. N refers to the number of countries.

**Table 6:** Dynamic panel threshold results—controlling for WHO regions (continue).

Variable	European Region			South-East Asian Region			Western Pacific Region		
	Head-Count	P-Gap	P-Gap^2^	Head-Count	P-Gap	P-Gap^2^	Head-Count	P-Gap	P-Gap^2^
Threshold (γ)	2.331 ***	3.444 ***	2.887 ***	2.661 ***	3.028 ***	2.661 ***	2.121 ***	1.024 ***	1.426 ***
	−0.087	−0.147	−0.127	−0.275	−1.052	−0.165	−0.03	−0.19	−0.182
Out-of-pocket									
${\hat{\beta }}_{1}$	18.631 **	1.618 **	17.173 ***	−2.641	−0.626	−6.345	1.664 ***	2.241 ***	1.673 ***
	−7.484	−0.789	−3.397	−6.447	−3.617	−4.212	−0.533	−0.237	−0.333
${\hat{\beta }}_{2}$	−16.971 **	4.002 ***	−15.514 ***	3.345	3.676	6.419	−2.399 ***	−2.106 ***	−1.570 ***
	−7.419	−1.422	−3.377	−5.437	−8.658	−3.996	−0.909	−0.435	−0.543
$Pov_{it-1}$	0.484 ***	0.540 ***	0.545 ***	0.958 **	0.648	1.068 **	−0.200	0.515 ***	0.564 ***
	−0.039	−0.043	−0.045	−0.439	−0.519	−0.497	−0.163	−0.03	−0.038
$GDP_{it}$	−1.197 ***	−1.230 ***	−1.049 ***	−0.743	−1.549	−0.490	−3.835 ***	−1.243 ***	−1.320 ***
	−0.198	−0.287	−0.279	−0.892	−1.035	−1.562	−0.447	−0.135	−0.219
$GHE_{it}$	0.663 ***	1.251 ***	0.436 ***	0.214	0.098	0.172	−0.312 **	0.043	0.366
	−0.159	−0.256	−0.345	−1.06	−1.214	−1.295	−0.13	−0.178	(0.226)
Upper regime (%)	95.3	33.7	70.5	94.4	81.5	94.4	75	87.04	82.4
Bootstrap (*p*-value)	0.5	0.3	0.9	0	0	0	0	0	0
N	145	145	145	145	145	145	145	145	145
No. moment conditions	32	32	32	32	32	32	32	32	32

Note: ***, ** denote 1%, 5%, respectively. Between ( ) are robust standard errors. N refers to the number of countries.

## Data Availability

The data presented in this study are openly available in WHO and World Bank databases.

## Appendix A

**Table A1:** IPS panel unit root test.

Variable	Level	First-Difference
$PHC_{it}$	−1.425	−2.937 ***
$PG_{it}$	−1.080	−2.223 ***
$PG_{it}^{2}$	−1.207	−2.425 ***
$OOP_{it}$	−1.268	−2.509 ***
$GDP_{it}$	−1.263	−2.460 ***
$GHE_{it}$	−1.661	−3.294 ***

Note: *** denotes 1% significance level. The first-difference critical values are −1.770, −1.840, and −2.000 at 10%, 5%, and 1%, respectively.

**Table A2:** Robustness analysis dynamic panel threshold results—all countries.

Variable	Head-Count	P-Gap	P-Gap^2^
Threshold (γ)	3.399 ***	3.399 ***	3.399 ***
	(0.194)	(0.175)	−0.169
Out-of-pocket			
${\hat{\beta }}_{1}$	−0.444	−0.480	−0.549
	(0.356)	(0.409)	(0.499)
${\hat{\beta }}_{2}$	1.567 **	1.704 **	1.928 **
	(0.656)	(0.754)	(0.838)
$Pov_{it-1}$	0.280 ***	0.286 ***	0.272 ***
	(0.067)	(0.065)	(0.067)
$GDP_{it}$	−1.929 ***	−2.056 ***	−2.241 ***
	(0.190)	(0.246)	(0.294)
$GHE_{it}$	−0.038	−0.037	−0.046
	(0.062)	(0.067)	(0.084)
$COR_{it}$	0.040	0.005	−0.043
	(0.032)	(0.043)	(0.054)
$GINI_{it}$	−0.032	−0.028	−0.031
	(0.028)	(0.034)	(0.038)
Upper regime (%)	55	55	55
Bootstrap (*p*-value)	0.08	0.04	0.34
N	145	145	145
No. moment conditions	40	40	40

Note: ***, ** denote 1%, 5%, respectively. Between ( ) are robust standard errors. N refers to the number of countries. The variables COR and GINI refer to corruption index (International Country Risk Guide) and gini coefficient, respectively.

**Table A3:** List of countries.

Algeria	Cameroon	Liberia	Russian Federation
Angola	El Salvador	Lithuania	Rwanda
Argentina	Estonia	Luxembourg	Samoa
Armenia	Eswatini	Madagascar	Senegal
Australia	Ethiopia	Malawi	Serbia
Austria	Fiji	Malaysia	Seychelles
Bangladesh	Finland	Maldives	Sierra Leone
Belgium	France	Mali	Slovakia
Belize	Gabon	Malta	Slovenia
Benin	Gambia	Mauritania	Solomon Islands
Bhutan	Georgia	Mauritius	South Africa
Bolivia	Ghana	Mexico	Spain
Bosnia and Herzegovina	Guatemala	Micronesia	Sri Lanka
Botswana	Guinea	Mongolia	Sudan
Brazil	Guinea-Bissau	Morocco	Suriname
Bulgaria	Guyana	Mozambique	Sweden
Burkina Faso	Haiti	Myanmar	Switzerland
Burundi	Honduras	Namibia	Tajikistan
Cabo Verde	Hungary	Nepal	Thailand
Canada	Iceland	Netherlands	Togo
Central African Republic	India	Nicaragua	Tonga
Chad	Indonesia	Niger	Trinidad and Tobago
Chile	Iran (Islamic Republic of)	Nigeria	Tunisia
China	Ireland	Norway	Turkey
Colombia	Israel	Pakistan	Turkmenistan
Comoros	Italy	Panama	Tuvalu
Congo	Jamaica	Papua New Guinea	Uganda
Costa Rica	Japan	Paraguay	Ukraine
Croatia	Jordan	Peru	United Kingdom
Czechia	Kazakhstan	Philippines	United Republic of Tanzania
Côte d’Ivoire	Kenya	Poland	United States of America
Democratic Republic of the Congo	Kiribati	Portugal	Uruguay
Denmark	Kyrgyzstan	Republic of Korea	Uzbekistan
Dominican Republic	Lao People’s Democratic Republic	Republic of Moldova	Vanuatu
Ecuador	Latvia	Republic of North Macedonia	Venezuela
Egypt	Lesotho	Romania	Viet Nam
			Zambia
